# A multiscale optimisation method for bone growth scaffolds based on triply periodic minimal surfaces

**DOI:** 10.1007/s10237-021-01496-8

**Published:** 2021-07-27

**Authors:** E. F. Lehder, I. A. Ashcroft, R. D. Wildman, L. A. Ruiz-Cantu, I. Maskery

**Affiliations:** Centre for Additive Manufacturing, Faculty of Engineering, The University of Nottingham, Nottingham, NG7 2RD UK

**Keywords:** Bone scaffolds, Minimal surfaces, Additive manufacturing, Tissue regeneration, Level set method, Multi-scale modelling

## Abstract

Tissue engineered bone scaffolds are potential alternatives to bone allografts and autografts. Porous scaffolds based on triply periodic minimal surfaces (TPMS) are good candidates for tissue growth because they offer high surface-to-volume ratio, have tailorable stiffness, and can be easily fabricated by additive manufacturing. However, the range of TPMS scaffold types is extensive, and it is not yet clear which type provides the fastest cell or tissue growth while being sufficiently stiff to act as a bone graft. Nor is there currently an established methodology for TPMS bone scaffold design which can be quickly adopted by medical designers or biologists designing implants. In this study, we examine six TPMS scaffold types for use as tissue growth scaffolds and propose a general methodology to optimise their geometry. At the macro-scale, the optimisation routine ensures a scaffold stiffness within suitable limits for bone, while at the micro-scale it maximises the cell growth rate. The optimisation procedure also ensures the scaffold pores are of sufficient diameter to allow oxygen and nutrient delivery via capillaries. Of the examined TPMS structures, the Lidinoid and Split P cell types provide the greatest cell growth rates and are therefore the best candidates for bone scaffolds.

## Introduction

Standard procedures for the repair of critical bone defects or fractures are bone allografts, where the graft is from a donor, and autografts, where it is from the patient. The latter option is generally preferable as it presents a lower risk of tissue rejection and disease transmission (Zimmermann and Moghaddam 2011). However, there are several drawbacks to these processes: limited material availability, long surgical operation time (as bone is removed then re-implanted), blood loss and pain, as well as potential complications at the donor site (Wang and Yeung 2017). Synthetic graft materials such as calcium phosphate (CaP), tricalcium phosphate (TCP) and hydroxyapatite possess mechanical properties similar to those of the organic part of bone, making them a possible alternative. However, for cells to migrate, attach, proliferate and differentiate into bone tissue, an adequate bone-mimicking interconnected structure is required (Ma et al. 2018), and this cannot easily be achieved with traditional manufacturing techniques. Additive manufacturing (AM) enables accurate control of the scaffold geometry and microstructure, which potentially results in scaffolds with superior pore interconnectivity and improved mechanical properties relative to those created using traditional methods (Jariwala et al. 2015).

Scaffolds based on triply periodic minimal surfaces (TPMS) are attractive for bone tissue engineering because their porosities are easily tuneable to match functional requirements (Vijayavenkataraman et al. 2018), they have been shown to yield a scaffold structural stiffness close to that of bone (Shi et al. 2018), and because they possess high surface-to-volume ratio, thus enabling more cell attachment compared to other geometries (Vijayavenkataraman et al. 2018). Various studies have examined the effect of TPMS type on scaffold properties such as porosity, pore size, stiffness and curvature (Eglin et al. 2017; Vijayavenkataraman et al. 2018) but structure-property relationships relating to cell growth in TPMS scaffolds have not been developed to date. This study provides such structure-property relationships for several of these scaffolds as well as a new optimisation method for bone scaffold design.

The in vitro study of Rumpler et al. (2008) yielded two important results concerning the growth of pre-osteoblast cells on the surface of scaffolds: (a) the rates of bone tissue and cell growth increased with increasing concave curvature, and (b) very little bone tissue and cell growth was observed on planar and convex surfaces, until the local environment became concave due to cell growth from other areas. Bidan et al. (2012) presented a model in which the dependence of cell and tissue growth on geometrical features was due to mechanical forces at the surface of the scaffold. This same model was used by Gamsjger et al. (2013) and they say that pre-osteoblast cells grow faster on concave surfaces because of, “the presence of contractile tensile stresses produced by cells near the tissue surface” (Gamsjger et al. 2013). The model predictions agreed well with results from 2D scaffolds, including those of Rumpler et al. (2008).

A useful structure-property model for bone scaffold design would link the cell growth rate to some controllable geometrical property (e.g. curvature). Such a model was presented by Guyot et al. (2014), where the relationship between surface curvature and the rate of pre-osteoblast growth (derived for tissue growth in 2D scaffolds (Rumpler et al. 2008)) was combined with the level set method to accurately predict cell growth in 3D porous scaffolds (Guyot et al. 2014). Guyot et al. (2014) tested for pre-osteoblast cells in their study, and thus, since our work is based partly on their model, we refer only to cell growth rather than tissue growth throughout this paper.

Long bone fractures are some of the most common injuries in the musculoskeletal system (Pivonka and Dunstan 2012). When these fractures are 25 mm or longer, they are deemed “critical” as they do not heal unaided (Schemitsch 2017). Such nonunion fractures are particularly common in the femur of adults (Ma et al. 2016). Femoral critical fractures were therefore chosen to act as a test case in this study, both because of how common critical fractures at this site are and because they are representative of other long bone critical fractures. Internal fracture fixation plates made of biologically inert metals are the most stable fixation devices to aid in the healing of critical long bone fractures (Lee et al. 2016; Uhthoff et al. 2006). These plates are often used in combination with bone allografts or autografts. In this study, we assume a fracture fixation plate is to be used together with the proposed scaffold as this affects the allowable scaffold stiffness range.

In this study, we used the level set model introduced by Guyot et al. (2014) in combination with mechanical performance and pore size analysis to predict and optimise the performance of TPMS bone scaffolds for femoral fractures. The process consisted of a volume fraction optimisation where the property to be maximised was the average growth rate of pre-osteoblast cells. The optimisation constraints were based on the axial stiffness and the pore size of the scaffolds (which must be sufficient to allow oxygen and nutrient delivery via capillaries). The aim of the study was to develop a methodology to select the optimal lattice type and volume fraction for a bone scaffold. Such a method is needed to ensure a chosen scaffold design will provide the fastest healing rate. Our proposed design method provides a distinct advantage over previous methods in that it allows for a very clear graphical representation of the constrained solution space. Our motivation stems from the big gap between biomedical engineering research and the actual implementation of that work for practical applications, which often comes from researchers not taking into consideration the adoption of the technology by biologists designing implants or medical designers (O’Donnell et al. 2019). Moreover, the proposed stiffness and pore size models have not been used for this purpose before, so this work represents an advancement in the use of computational design and analysis methods for biomedical implants.

## Methods

### Scaffold optimisation

**Fig. 1:**
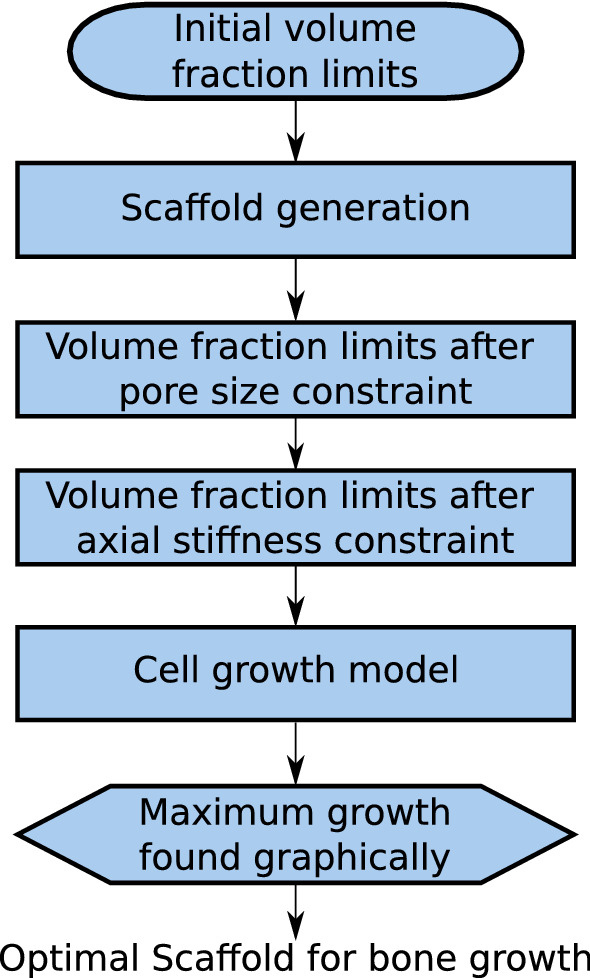
Schematic of the graphical multi-scale optimisation strategy

General optimisation problems are defined by an objective function, design variables and constraints, and are usually solved with iterative algorithms subject to some convergence criterion. In this work, the geometry of a TPMS scaffold of fixed size was defined by two design variables: the TPMS type and the volume fraction. The objective function to be maximised was the pre-osteoblast cell growth rate, subject to pore size and stiffness constraints. The optimisation method therefore benefits from ease of graphical representation, which is a strong motivator for its use, as it allows for the clear correlation between lattice design variables and performance (i.e. stiffness and cell growth). This is crucial if the optimisation method is to be translated into implementable design rules for designers of bone growth scaffolds, where the ability to ‘tune’ the performance of a scaffold, for example, its stiffness, allows for the creation of patient- and fracture-specific designs to provide minimal stress-shielding. Our optimisation method is illustrated in Fig. 1.

First, a scaffold cell size of 1 mm was selected. Scaffold size could be considered a third design variable, but it was fixed here in order to develop a graphical, easily implementable optimisation method for TPMS type and volume fraction. These have been shown, unlike scaffold size, to have a significant effect on both scaffold stiffness and cell growth rate (Diez-Escudero et al. 2020; Maskery et al. 2018a). This allowed for the selection of suitable volume fraction limits, as explained in Sect. 3.1. After this, the TPMS scaffold geometry was generated using the method of Maskery et al. (2018b), as described in Sect. 2.2. This was followed by applying pore size and stiffness constraints, as described in Sects. 2.3 and 2.4, respectively. Applying these constraints provides the viable volume fraction ‘window’ for each TPMS scaffold type. The final step was to determine which volume fraction corresponds to the maximum pre-osteoblast cell growth rate for each scaffold type. This was carried out using the level set cell growth model described in Sect. 2.5.

### Scaffold generation

The scaffold types used for this study are based on triply periodic minimal surfaces (TPMS). We examined six available TPMS scaffold types: Primitive, Gyroid, Split P, Diamond, Lidinoid and Neovius. The Gyroid, Diamond, Primitive and Neovius are among the most commonly studied TPMS types (Han and Che 2018), while the other types were chosen due to their large surface-to-volume ratios and high local curvatures, both of which promote rapid cell growth (Abueidda et al. 2017). The surface equations used to generate these scaffold types share the terms presented in Eqs. 2 and 3 (Maskery et al. 2018b). $k_{i}$ are the TPMS periodicities;

1 $$\begin{aligned} k_i=2\pi n_i, \end{aligned}$$

where $i = x, y, z$ and $n_{i}$ are the numbers of cell repetitions in each direction in the resulting scaffolds. The following terms are shorthand notations for sine and cosine expressions:

2a $$\begin{aligned}&S_{i}={\rm sin}\Big (k_i \frac{i}{L_i}\Big ), \end{aligned}$$

2b $$\begin{aligned}&S_{2i}={\rm sin}\Big (2k_i \frac{i}{L_i}\Big ), \end{aligned}$$

2c $$\begin{aligned}&C_{i }={\rm cos}\Big (k_i \frac{i}{L_i}\Big ), \end{aligned}$$

2d $$\begin{aligned}&C_{2i}={\rm cos}\Big (2k_i \frac{i}{L_i}\Big ), \end{aligned}$$

where $L_{i}$ are the absolute sizes of the scaffold in the three orthogonal directions. The $U=0$ isosurface is then found from:

3a $$\begin{aligned}&U_{\rm Primitive}=\Big (C_x+C_y+C_z\Big )^2-t^2, \end{aligned}$$

3b $$\begin{aligned}&U_{\rm Gyroid}=\Big (S_xC_y+S_yC_z+S_zC_x\Big )^2-t^2, \end{aligned}$$

3c $$\begin{aligned}&U_{\rm Split P}=\Big (1.1\big (S_{2x}C_yS_z+S_{2y}C_zS_x+S_{2z}C_xS_y\big )- \nonumber \\&\qquad \qquad 0.2\big (C_{2x}C_{2y}+C_{2y}C_{2z}+C_{2z}C_{2x}\big )- \nonumber \\&\qquad \qquad 0.4\big (C_{2x}+C_{2y}+C_{2z}\big )\Big )^2-t^2, \end{aligned}$$

3d $$\begin{aligned}&U_{\rm Diamond}=\Big (C_xC_yC_z+S_xS_yS_z+S_xC_yS_z+C_xS_yS_z\Big )^2-\nonumber \\&\qquad \qquad \quad {}t^2, \end{aligned}$$

3e $$\begin{aligned}&U_{\rm Lidinoid}=\Big (\big (S_{2x}C_yS_z+S_{2y}C_zS_x+S_{2z}C_xS_y\big )-\nonumber \\&\qquad \qquad \;\;\,\big (C_{2x}C_{2y}+C_{2y}C_{2z}+C_{2z}C_{2x}\big )\Big )^2-t^2, \end{aligned}$$

3f $$\begin{aligned}&U_{\rm Neovius}=\Big (3\big (C_x+C_y+C_z\big )+4C_xC_yC_z\Big )^2-t^2, \end{aligned}$$

where t is an arbitrary parameter used to control the volume fraction of the resulting scaffold (Maskery et al. 2018a), which is the fraction of the scaffold bounding volume that consists of material. The $U=0$ isosurface is then treated as a boundary between solid and void domains of the scaffold. This was followed by a voxelisation of the solid region to apply the cell growth model. These voxel models were then translated into hexahedral finite element meshes for assessment of the modulus of the scaffold under compressive loading. Figure 2 shows the eight TPMS lattice types used. For a thorough understanding of the scaffold generation process, see the work of Maskery et al. (2018a, 2018b).

**Fig. 2:**
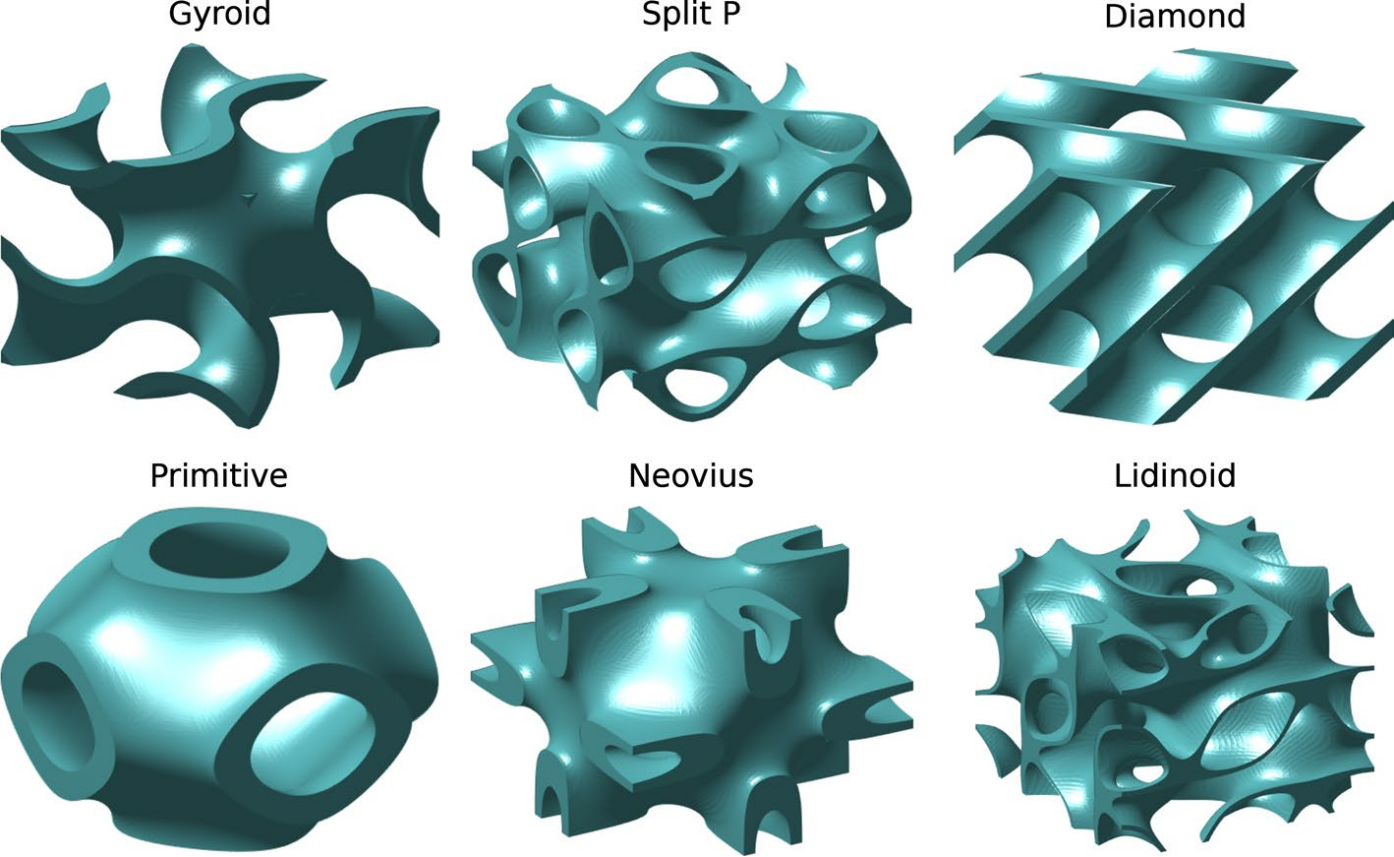
The six triply periodic minimal surface (TPMS) geometries used in this study. All at 0.2 volume fraction

### Pore size constraint

It was previously found that 100 μm is the minimum pore size diameter that allows for capillary infiltration into the scaffold in vivo (Bruauskait et al. 2016; Lim et al. 2019). This is due to the diameter of capillaries that must populate the scaffold to provide oxygen and nutrients for cell survival (Lim et al. 2019). Additionally, several studies have shown that the diffusion limit of oxygen and nutrients is 200 μm (Carmeliet and Jain 2000), so it follows that cell growth on scaffold surfaces may be inhibited if they are separated by 200 μm from a pore. Therefore, scaffold pores should have a diameter of at most 400 μm so that the scaffold may become fully populated with cells. Thus, we defined the maximum and minimum allowed pore diameters to be 400 μm and 100 μm, respectively. It should be noted that these pore size limits are different for scaffolds where the cells are encapsulated within solid scaffold walls, as opposed to residing at the surface (Rouwkema et al. 2013).

**Fig. 3:**
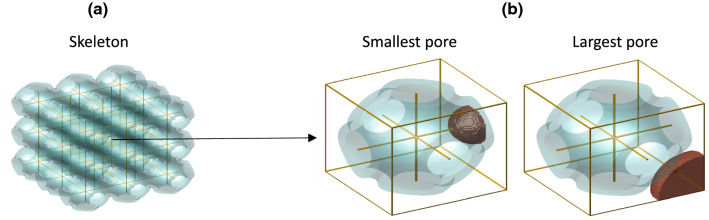
Computation of smallest and largest pores in the scaffolds. **a** The medial skeleton is computed using a $3 \times 3 \times 3$ cell TPMS scaffold. **b** The minimum and maximum pores are calculated using a distance function

The minimum and maximum pore sizes of TPMS scaffolds were found by first determining the medial axis skeleton of the void domain with a method adapted from that of Kerschnitzki et al. (2013) to measure the position of minerals within a porous network (Kerschnitzki et al. 2013). For each scaffold type, this was done using a voxel representation of a $3 \times 3 \times 3$ unit cell scaffold, which is sufficient to ensure that the largest and smallest void volumes are included in the analysis. An illustration of the medial axis skeleton is shown in Fig.3a. A distance function (Maurer et al. 2003) was then computed for every part of the medial skeleton and every voxel in the solid scaffold domain, giving the minimum and maximum sizes of virtual spheres that could sit inside the scaffold’s empty space (see the examples in Fig.3b). The diameters of these spheres were taken to represent the minimum and maximum pore size for each scaffold.

### Axial stiffness constraint

**Fig. 4:**
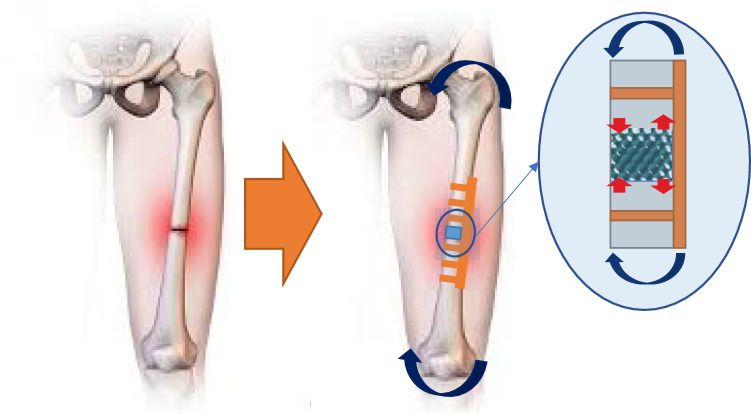
On the right is a fractured femur implanted with both a fixation plate and scaffold. Moments and loads are shown by arrows. Axial stiffness for a 30 mm gap needs to be between 1000 and 2700 N/mm (Steiner et al. 2014). The image on the left shows the original artwork from (Gersony 2000)

An optimal bone scaffold should possess sufficient stiffness to avoid refracture under loading. For the femur, the critical loading is axial along the length of the bone (Duda et al. 1997). The fracture fixation plate (shown in Fig.4) may be designed to provide sufficient stiffness, but stiff plates lead to bone resorption under the plate through stress shielding (Claes and Heigele 1999). However, the scaffold cannot be too stiff either because the bone interfragmentary movement (IFM) (Claes and Heigele 1999), which refers to the movement between the fractured bone fragments in the axial direction, must be above a minimum value. This is necessary for the bone cells to experience sufficient strain for bone formation. It follows that there is a minimum allowable scaffold stiffness as well as a maximum. A suitable range of axial stiffness for a bone fracture of 30 mm was defined by Steiner et al. (2014) to be between 1000 and 2700 N/mm (Steiner et al. 2014).

For a cylindrical scaffold of diameter *D* and height *L*, the axial stiffness, $k_{\rm scaff}$, is

4 $$\begin{aligned} {k_{\rm scaff}=E^*E \frac{\pi D^2}{4L},} \end{aligned}$$

where $E^*$ is a dimensionless factor known as the relative modulus and *E* is the elastic modulus of the scaffold material. The material was assigned the modulus of additively manufactured Nylon, 1.8 GPa. Nylon was selected as a model material because it has similar mechanical properties to trabecular bone (Wu et al. 2018) which has been shown to be beneficial as it allows the scaffold to act as a woven-bone surrogate for lamellar bone (Reznikov et al. 2019). Nylon has been previously used to create additively manufactured non-degradable scaffolds for bone regeneration and showed higher bone ingrowth compared with the standard material, titanium, in a sheep femur bone defect (Reznikov et al. 2019).

We obtained general Gibson–Ashby scaling laws (Gibson et al. 2010) relating $E^*$ to the scaffold volume fraction, $\rho ^*$, using the same finite element (FE) approach as Maskery et al. (2018a). Compressive loading was applied to the top surfaces of FE scaffold models, and the reaction force and displacement were used to calculate the modulus. This was done for each scaffold type in this study (i.e. those originating from Eq. 3) and for a range of volume fractions from 0.2 to 0.9. The resulting moduli were fit with Gibson-Ashby laws of the form:

5 $$\begin{aligned} E^*(\rho ^*)=C_1\rho ^{*n}+E_0^*, \end{aligned}$$

where the parameters $C_1$, n and $E_0$ differ for each scaffold type. The determined parameters for several scaffold types are given in Table 1. These were selected from the full range of scaffold types due to their particular relevance to the scaffold optimisation method in Sect. 3.1. The parameters in Table 1, along with values for *D* and *L*, were used to predict $k_{\rm scaff}$ for each scaffold type. *D* was given the value 30 mm, the diameter of the femur, and *L* was 30 mm, the length of a critical bone fracture.

**Table 1:** Gibson–Ashby scaling parameters

	$C_1$	*n*	$E_0^*$
Split P	1.33	2.04	− 0.078
Gyroid	1.33	2.68	− 0.002
Diamond	1.26	2.74	0.039
Lidinoid	1.38	2.59	− 0.050

### Cell growth model

A computational model for pre-osteoblast cell proliferation was developed based on the work of Guyot et al. (2014). Cell proliferation is represented here as an advancing surface which grows from the original solid scaffold into the void domain. Guyot et al. (2014)’s work included validation of the level set model with experimental observation (Guyot et al. 2014) and was found to be representative of cell growth in a cell-seeded bone regeneration scaffold. The model implemented here is particularly convenient because, by using the level set method, it can be applied to any 3D geometry, not just TPMS scaffolds. In this study, we used a finite difference method, while Guyot et al. (2014) used a finite element method, hence a validation study is presented in “Appendix A” showing that the two implementations yield similar results. We used the same time step as in the study by Guyot et al. (2014), $10^{-4}$.

For each scaffold, a 3D distance function, $\varphi$, is calculated through a defined series of time steps, *t*. The $\varphi =0$ isosurface is an interface which advances from the original solid scaffold into the available empty space (the pores), as given in the equation as follows:

6 $$\begin{aligned} {\frac{\delta \varphi }{\delta t}+u\cdot \nabla \varphi =0,} \end{aligned}$$

The rate of advance of the $\varphi =0$ interface is the advection velocity, *u*;

7 $$\begin{aligned} u = {\left\{ \begin{array}{ll} -kn &{}\hbox { if k}\ >0\\ 0 &{}\hbox { if k}\ \le 0 \end{array}\right. } \end{aligned}$$

which is proportional to the local curvature, *k*;

8 $$\begin{aligned} {k=\nabla \cdot n,} \end{aligned}$$

In turn, *k* is calculated at each time step and is proportional to the normal of the interface denoted by *n*;

9 $$\begin{aligned} {n=\frac{\nabla \varphi }{|\nabla \varphi |},} \end{aligned}$$

**Fig. 5:**
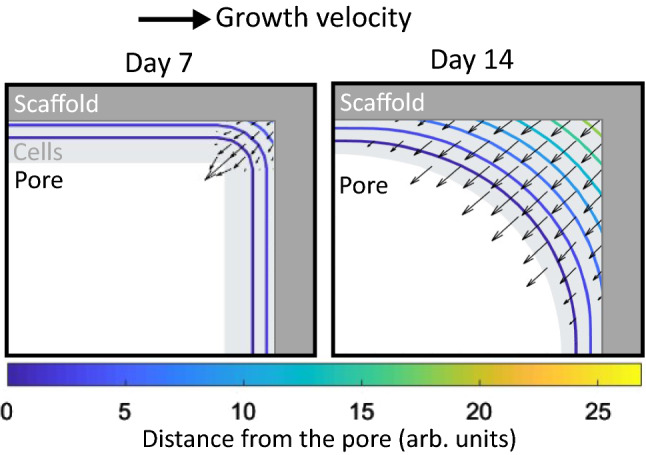
The principle of the cell growth model. On the left (day 7) is a thin layer of cells attached to the scaffold with contours showing the distance from the pore. Arrows show the growth velocity with both magnitude (length of arrow) and direction. On the right (day 14), the cells have proliferated to form a roughly circular pore

The cumulative cell growth at any time is given by the difference in volume between the $\varphi =0$ interface at that time, and the original scaffold. An illustration of this model applied in a simple 2D case is shown in Fig. 5; the local curvature due to the corner of the original scaffold drives rapid cell growth.

A mesh convergence analysis was performed to determine the number of voxels required for accurate cell growth modelling. The cell growth rate was calculated for scaffolds discretised into increasing numbers of voxels, from 125,000 up to 15.625 million. The total number of voxels was deemed appropriate when the absolute change in cell growth rate between successive discretisation values was lower than $1\%$. Scaffolds with one million voxels satisfied this criterion and were therefore used for cell growth modelling throughout this study.

## Results

### Pore diameter and stiffness constraints

**Fig. 6:**
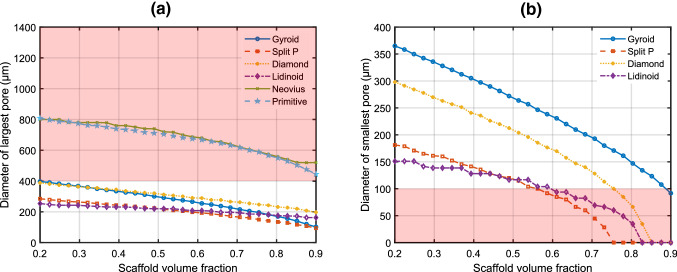
**a** the diameter of the largest pore for all scaffold types. **b** the diameter of the smallest pore of the five scaffold types that satisfied the maximum pore size constraint. The shaded areas indicate regions outside of the allowable range of pore sizes. All scaffold cells have dimensions of $1\times 1\times 1$ mm

As discussed in Sect. 2.3, an optimal bone scaffold must satisfy pore size constraints determined by the delivery of oxygen and nutrients to the growing cells. Figure 6a shows that while the Lidinoid, Split P, Diamond and Gyroid scaffold types have a maximum pore size below the limit of 400 $\mu$m for some of the volume fraction range, the Primitive and Neovius scaffold types do not satisfy this constraint and were therefore discarded from the study. The smallest pore size constraint in Fig.6b eliminates some of the volume fraction range for the Gyroid, Split P, Diamond and Lidinoid types.

**Fig. 7:**
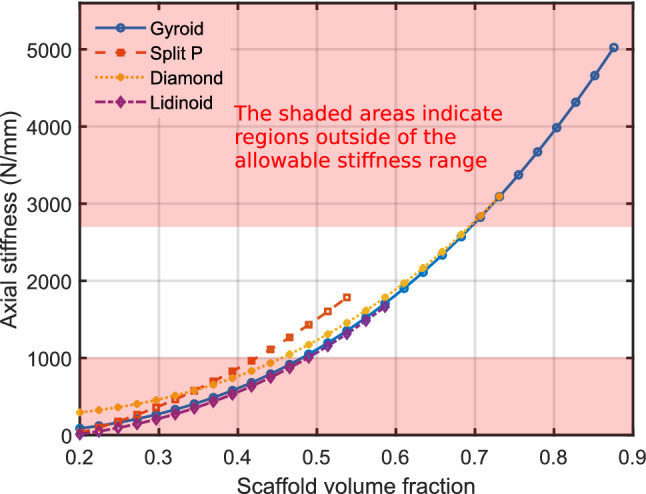
Applying pore limits to stiffness data. The shaded areas indicate regions outside of the allowable stiffness range. The application of stiffness limits is not shown here but is given in Table 2

Based on the pore size analysis, the volume fraction limits for the remaining scaffold types were updated. Figure7 illustrates the application of these limits to the stiffness data from FE compressive loading models.

In the next optimisation step, any scaffold design that did not provide an axial stiffness in the range 1000–2700 N/mm was discarded. As described in the methodology, these constraint ensure appropriate healing. Table 2 provides the final minimum and maximum limits for the allowed volume fraction taking both allowable cell pore size and stiffness ranges into account.

**Table 2:** Final volume fraction limits after applying axial stiffness constraints

	Minimum volume fraction	Maximum volume fraction
Gyroid	0.47	0.66
Split P	0.44	0.54
Diamond	0.44	0.66
Lidinoid	0.49	0.59

### Cell growth

**Fig. 8:**
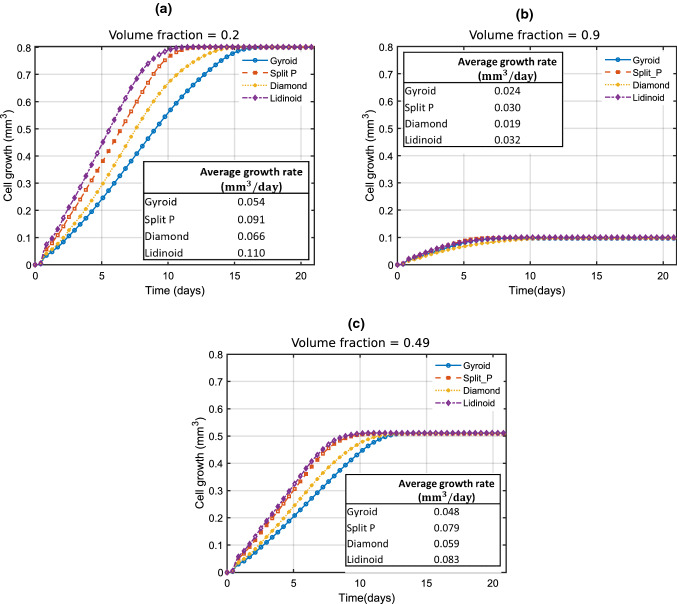
Cell growth after 21 days for scaffold volume fractions of 0.2, in (**a**), 0.9, in (**b**), and 0.49, in (**c**)

Figure 8a, b shows the cell growth predicted by the level set model detailed in Sect. 2.5 over a period of 21 days. Just three plots, for volume fractions of 0.2, 0.9 and 0.49, are shown here, but cell growth was calculated for the full range of volume fractions from 0.2 to 0.9.

**Fig. 9:**
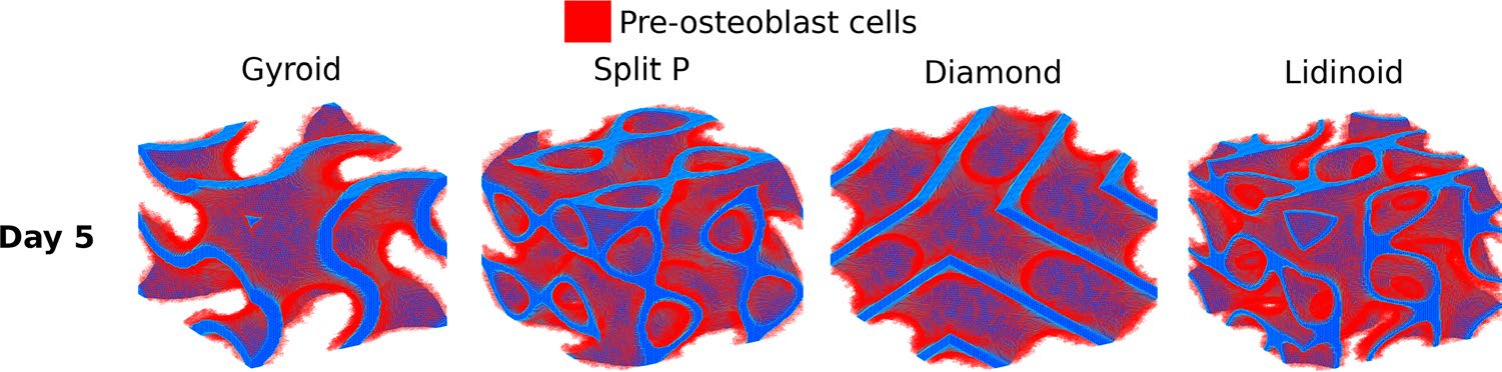
Pre-osteoblast cell growth after 5 days on various scaffold types, all at 0.2 volume fraction

The Lidinoid type yields the fastest cell growth for the entire volume fraction range, but the Split P type yielded a very similar growth curve at a volume fraction of 0.49 and 0.9. The average growth rate for each scaffold was calculated by dividing the volume of cells by the time taken for the cells to entirely fill the space of the scaffold which was initially empty, i.e. the time at which the curves in Fig. 8a, b plateaued. A visualisation of the cell growth throughout the surface of the scaffold is presented in Fig. 9.

**Fig. 10:**
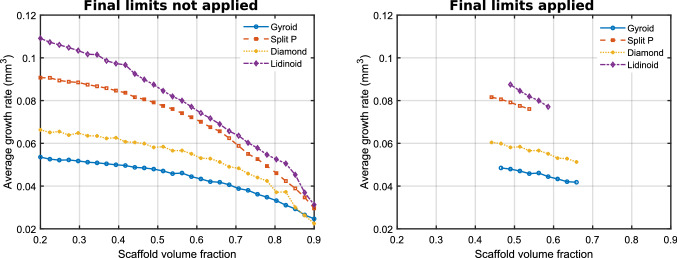
Average pre-osteoblast cell growth of each of the TPMS scaffold types after 21 culture days

For each volume fraction, the average growth rate was determined to compute the curves shown in Fig.10. At all volume fractions, the Lidinoid scaffold type provides the greatest average growth rate. In Fig.10, it can be observed that after removing the volume fractions that did not satisfy the stiffness and pore size constraints, the maximum average cell growth rates of the Split P and lidinoid scaffold types are very similar. Table 3 shows that the highest cell growth rate is predicted to be 0.0872 $\hbox {mm}^3$/day for the Lidinoid type. That was achieved with an optimal volume fraction of 0.49. However, the maximum growth rate for the Split P type was only $6\%$ lower.

**Table 3:** Optimal volume fractions after applying all constraints and the corresponding maximum cell growth rate

	Maximum cell growth rate ($\hbox {mm}^3$/day)	Optimal volume fraction
Gyroid	0.048	0.47
Split P	0.082	0.44
Diamond	0.060	0.44
Lidinoid	0.087	0.49

## Discussion

The proposed graphical optimisation method is a novel routine that can select the optimal scaffold type and volume fraction for a bone regeneration scaffold. The routine currently operates in a 2-variable solution space (the variables being scaffold type and volume fraction), which is sufficient to demonstrate this methodology. The optimisation routine is especially convenient because it enables a designer to visualise the entire solution space graphically and thus understand the scaffold selection criteria clearly and their impact on the responses. The optimisation procedure showed that out of the six initially available scaffold types, the Lidinoid scaffold with a volume fraction of 0.49 performed best with a cell growth rate that was 110% higher than that of the worst performing scaffold which satisfied the constraints.

By accounting for the minimum pore size limit of the scaffolds, the proposed optimisation routine ensured that blood capillaries can grow throughout the porous network. As described in Sect. 2.3, the proposed method to find the minimum pore size effectively calculates intersecting spheres throughout the entire porous network. Calculating the maximum pore size limit based on the diffusion of oxygen and nutrients from capillaries was also essential, as it ensured that the entire porous network could be filled with cells, thus allowing for a fair comparison between different TPMS scaffold types. This step includes the assumption that a capillary follows the medial path (or 1‘skeleton’) shown in Fig.3. There are two potential issues with this assumption. First, it excludes the possibility of more than one capillary passing through a given pore (Lim et al. 2019), and second, the capillaries might not always follow the medial path. The first issue does not invalidate the results of this study because two capillaries would facilitate greater oxygen delivery. The second issue is more of a concern because if the capillary is too far from a scaffold wall then the cells attached to that wall will not receive sufficient oxygen and die.

As explained in Sect. 2.4, mechanical stimulation of the scaffold and surrounding tissue also affects the bone growth outcome (Steiner et al. 2014), if there is too much strain, there will not be any growth, while small amounts of strain can be beneficial. The results of our study highlight the need for stiffness constraints, in the case of the Lidinoid cell type, these constraints reduced the allowable range of volume fractions by about 74%.

A micro-scale level set model was used to simulate the pre-osteoblast cell growth in TPMS scaffolds. The level set model has been validated with simpler geometries previously (Guyot et al. 2014). As explained in Sect. 2.5, the implementation used here was different to that used in the original study (Guyot et al. 2014), and although we have successfully validated our method (see “Appendix A”), we recommend that anyone interested in implementing this model also looks at the original implementation (Guyot et al. 2014). It was found that the average cell growth rate reduced consistently as the volume fraction was increased. The Lidinoid scaffold type not only yielded the maximum cell growth for its optimal volume fraction but also for the entire range of volume fractions. A recent study (Scerrato et al. 2021) presents a useful tool for understanding the interaction between bone and a bio-resorbable scaffold based on a viscoporoelastic model. While some numerical investigation is also done, the study does not propose a method to optimise the design parameters as we do here.

Although the stiffness and the pore size were the only constraints used here, other constraints can be easily added. An example would be the allowable shear stress caused by the surrounding fluids, which could be incorporated using the relationship between shear stress and cell growth discussed by Guyot et al. (2015). Another example would be looking at multi-material scaffolds, where the growth of capillaries could become a constraint. Such an approach could benefit from the work of Bednarczyk and Lekszycki (2016) who proposed a novel model for the growth of capillaries and nutrient supply. One more example is that of biodegradable scaffolds, where the scaffold degradation rate is chosen to complement the cell growth rate as suggested by Sanz-Herrera et al. (2009).

The methodology could also be adapted to use different design variables and growth models. Giorgio et al. (2020) discuss various interesting bio-inspired cellular scaffold geometries and they also reflect on the importance of considering the effects of the geometry at the micro-scale as well as the macro-scale. It could then be useful to use our methodology in order to optimise the micro- and macro-scale geometry using a multi-scale model with one design variable for each scale.

**Fig. 11:**
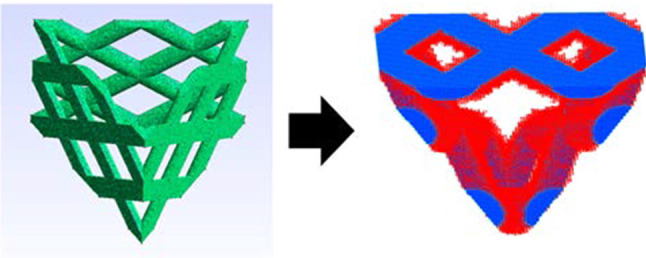
Tetrahedral mesh from Guyot et al. (2014) (left) and voxelised mesh used in this study as well as darker voxels showing cell growth after five days (right)

**Fig. 12:**
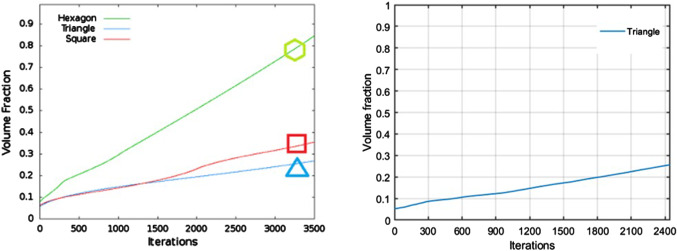
Model results from Guyot et al. (2014) (left) and those computed in this study (right). In both graphs, the number of iterations is equivalent to 16.3 days. Note that ‘Volume fraction’ in these figures refers to the cell growth as a fraction of the total volume enclosing the scaffold

The optimal Lidinoid TPMS geometry of this study outperforms both the 2D scaffolds in the study of Rumpler et al. (2008) as well as those in the study of Guyot et al. (2014). The maximum average cell growth rate achieved with our optimal scaffold was about 140% greater than that achieved by the 2D triangle scaffold in the study of Rumpler et al. (2008). When comparing our cell growth results with those of Guyot et al. (2014), where non-optimised 3D geometries are used (Figs. 11, 12), it can be seen that the maximum average cell growth rate achieved by the optimal Lidinoid scaffold of our study is about 90% higher than that achieved by the hexagon scaffold type of their study.

## Conclusion

The current paper demonstrates the design of optimal TPMS-based bone growth scaffolds combining computational analysis and a simple graphical framework which could be easily adopted by medical designers or biologists designing implants. Although the theory regarding curvature-dependent cell/tissue growth still requires more understanding, experiments have already shown how cell growth is affected by curvature in 3D scaffolds. Hence, the novel methodology presented in this study can now be used to design optimal scaffolds that outperform the state of the art. While the constraint models used might vary depending on the application of the scaffold, the presented procedure provides a flexible approach to apply any constraint with suitable limits. The results of this study also show that AM is now the way forward for bone grafts, given that the optimal geometries discussed in this study can only be made via AM.

## Figures and Tables

**Fig. 13:**
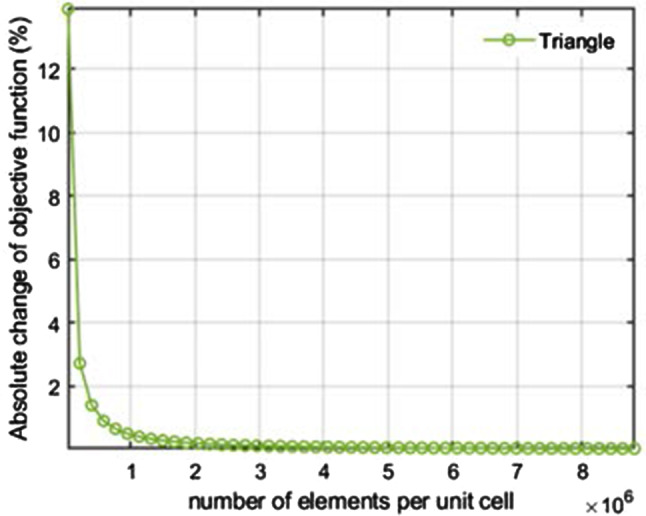
Mesh convergence for the triangular geometry. The objective function was the average growth rate and the *y*-axis on the plot represents the absolute change of this objective function in percentage. The *x*-axis shows the number of elements per scaffold unit cell

## Appendix A: Model validation and calibration

To validate the level set cell growth model used here, the triangular scaffold from the original cell growth study by Guyot et al. (2014) was used. The geometry was acquired from the authors of that work. The implementation described in Sect. 2.5 was used to compute the cell growth on the surface of the triangular scaffold, and the results were then compared with those provided by Guyot et al. (2014). The results are shown in Fig.12, where it can be seen that our prediction of cell growth for the triangular scaffold closely matches that of the original work (Fig. 12).

There are some differences in the curves, and it is hypothesised that these are because the original study used a tetrahedral mesh while here a hexagonal mesh is used. To ensure our result is correct, however, a mesh convergence study was performed (as shown in Fig. 13), and the cell growth rate was found to converge below a threshold of 1% at around 106 elements. For the purpose of this study, presenting a novel overall methodology to optimise bone regeneration scaffolds, this validation was deemed acceptable.
